# Simple sequence repeat variation in the *Daphnia pulex *genome

**DOI:** 10.1186/1471-2164-11-691

**Published:** 2010-12-03

**Authors:** Way Sung, Abraham Tucker, R Daniel Bergeron, Michael Lynch, W Kelley Thomas

**Affiliations:** 1 Hubbard Center for Genome Studies, University of New Hampshire, Durham, NH 03824, USA; 2 Department of Computer Science, University of New Hampshire, Durham, NH 03824, USA; 3 Department of Biology, Indiana University, Bloomington, IN 47405, USA

## Abstract

**Background:**

Simple sequence repeats (SSRs) are highly variable features of all genomes. Their rapid evolution makes them useful for tracing the evolutionary history of populations and investigating patterns of selection and mutation across gnomes. The recently sequenced *Daphnia pulex *genome provides us with a valuable data set to study the mode and tempo of SSR evolution, without the inherent biases that accompany marker selection.

**Results:**

Here we catalogue SSR loci in the *Daphnia pulex *genome with repeated motif sizes of 1-100 nucleotides with a minimum of 3 perfect repeats. We then used whole genome shotgun reads to determine the average heterozygosity of each SSR type and the relationship that it has to repeat number, motif size, motif sequence, and distribution of SSR loci. We find that SSR heterozygosity is motif specific, and positively correlated with repeat number as well as motif size. For non-repeat unit polymorphisms, we identify a motif-dependent end-nucleotide polymorphism bias that may contribute to the patterns of abundance for specific homopolymers, dimers, and trimers. Our observations confirm the high frequency of multiple unit variation (multistep) at large microsatellite loci, and further show that the occurrence of multiple unit variation is dependent on both repeat number and motif size. Using the *Daphnia pulex *genetic map, we show a positive correlation between dimer and trimer frequency and recombination.

**Conclusions:**

This genome-wide analysis of SSR variation in *Daphnia pulex *indicates that several aspects of SSR variation are motif dependent and suggests that a combination of unit length variation and end repeat biased base substitution contribute to the unique spectrum of SSR repeat loci.

## Background

Tandem arrays of DNA nucleotides, known as simple sequence repeats (SSR), are extremely dynamic parts of the genome. These tandem repeats vary in motif sequence, length, and repeat number. The most common SSR loci are homopolymers (repeated single nucleotide), dimers (repeated nucleotide pair), and trimers (repeated nucleotide triplet). The highly polymorphic nature of SSRs makes them desirable for use in both genotyping and population-level evolutionary studies. Simple sequence repeats may influence the fitness of the organism [1], and in specific cases are known to be causal of human disease [1].

A high mutation rate at SSRs has been well documented in a number of organisms using microsatellite constructs, pedigree analyses, and mutation accumulation (MA) experiments [2-4]. The high mutation rate in SSRs is due to a propensity for DNA misalignment during replication [5], regulated primarily by the universal mismatch repair system (MMR). MMR knockout experiments show dramatic increases (up to 100 fold greater) in the rate of simple sequence variation [6,7]; and suggest that surveillance by MMR may vary across the genome [8]. Although the MMR system has been well-categorized in certain species, the components of MMR may vary from species to species, which may result in lineage specific patterns of SSR repair, and consequently lineage specific patterns of SSR variation [9]. In addition to MMR fidelity; repeat number, motif sequence, motif size, local rates of recombination and genomic location can influence rates of SSR variation [8,10,11]. Genome wide analysis of SSR variation in well-characterized systems will facilitate a greater understanding of the relationship between MMR evolution, and the abundance of SSR and levels of variation at these loci.

*Daphnia pulex *(water flea), crustacean represents a particularly useful platform for the study of SSR evolution. The *Daphnia pulex *lineage is distinct from the two model organisms *Caenorhabditis elegans *(nematode) and *Drosophila melanogaster *(fruit fly), both of which have well characterized simple sequence repeat variation [2,12]. The genome sequence of *D. pulex *is based on a single heterozygous genotype sampled from a natural population with minimal inbreeding [13]. The heterozygosity within the genome sequence can be assayed by analysis of the raw sequence reads (8 × coverage) allowing a nearly genome-wide analysis of the variation at SSR loci.

There are two main goals of this study. The first is to provide a detailed catalog of SSR loci and their distribution within the genome of *Daphnia pulex*. The second goal is to assay SSR heterozygosity on a genome wide scale to test for motif specific rates and patterns of SSR evolution.

## Results and Discussion

### Catalog and Distribution of SSR loci

**Figure 1:**
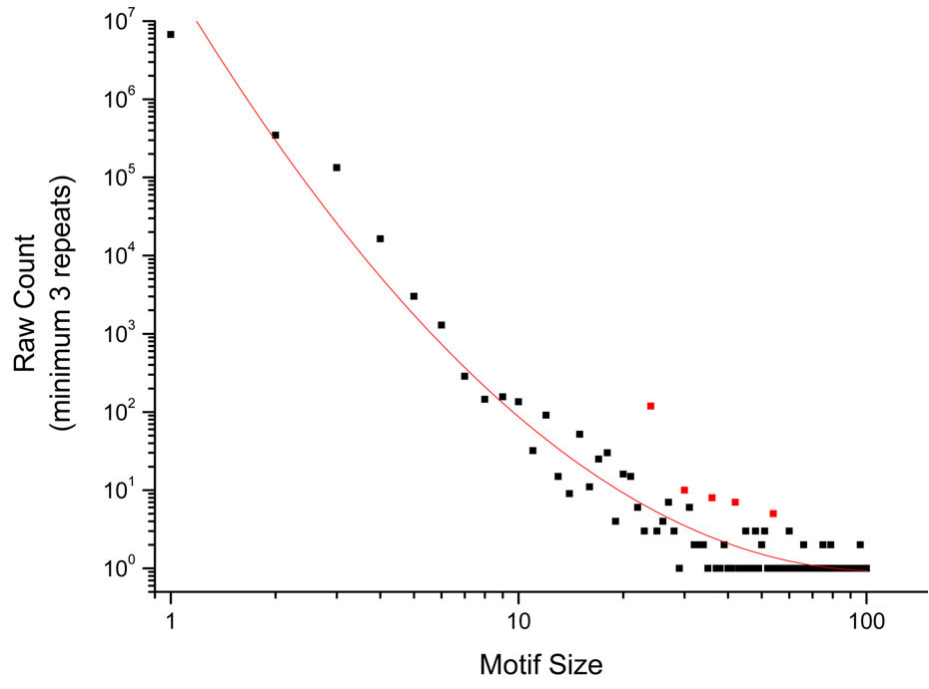
**Genome-wide *Daphnia pulex *count for simple sequence repeats for motif size 1-100**. Each simple sequence repeat has a minimum repeat size of 3, red points contains repeated amino acid motifs (r^2 ^= 0.957, p < 1e-10).

**Figure 2:**
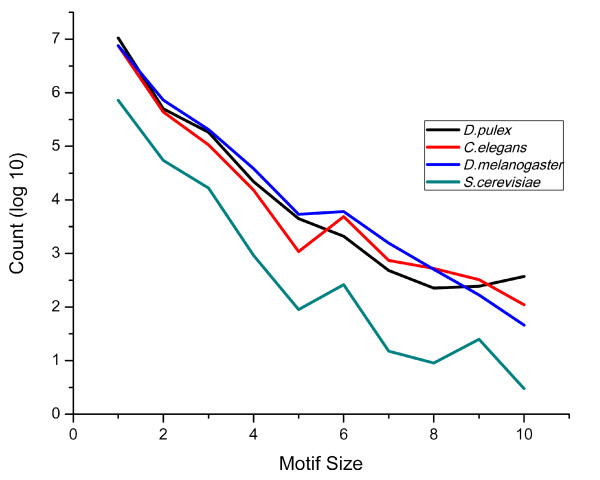
**SSR characteristics for motif sizes 1-10 with minimum 3 repeats in *Daphnia pulex*, *Caenorhabditis elegans*, *Drosophila melanogaster*, *Saccharomyces cerevisiae***.

In order to enumerate all types of SSRs in the *Daphnia pulex *genome, we first identified all SSR loci in the largest 100 scaffolds (*Daphnia pulex *assembly 9/01/2006; N50 = 103) with repeat motifs from 1 to 100 nucleotides, repeated perfectly three or more times. Motif size is defined by the length of the set of nucleotides that are repeated, while repeat number reflects the number of times that set is repeated. For example, the nucleotides ATATAT have a motif size of 2 (AT) and the number of repeats is 3. Under these criteria, we identified a total of 7,229,342 perfect SSRs, spanning 48.4 Mbp (21.3%) of the *Daphnia pulex *genome (Figure 1). As with all prior studies, the abundance of all SSR types exceeds random expectations based on nucleotide composition [14]. Homopolymeric repeats (HPs) make up 93% of all SSRs (6726771 loci), followed distantly by dimers (4.8%; 347288 loci) and trimers (1.8%; 133428 loci). The remaining SSRs with motifs larger than 3 base pairs constitute a much smaller fraction of the genome. The distribution of SSR in the *Daphnia pulex *genome most closely resembles the SSR distribution in *Caenorhabditis elegans *(93% HP, 5.3% dimers, 1.3% trimers) (Figure 2).

The number of simple sequence repeats follows an exponential decay with increasing motif size (Figure 1). The motifs with the size class of 24, 36, 42, 54, and 60 deviating from the regression line (Red in Figure 1). Closer analysis of these motif classes using *D. pulex *genome annotations [15] reveals that the overrepresented motifs are protein coding sequences of tandemly repeated amino acid motifs found in multiple copies that are spread across the genome. For example, the 24mers are multiple copies of an 8 amino acid repeat found in the largest subunit of DNA-directed RNA polymerase II. This inflates the count of these motifs, all of which are necessarily divisible by one codon unit.

### Abundance of Specific SSR Motif Types

The number of A/T homopolymers (5,276,283) vastly exceeds the number of G/C homopolymers (1,450,489). The ratio of A/T to G/C homopolymers is not predicted by the *Daphnia pulex *base composition (A/T 59%, G/C 41%, p = 0). The overabundance of A/T homopolymer tracts may reflect unique origins of the A/T homopolymers from A/T rich transposable elements [16,17] or base specific mutational patterns in repetitive sequence motifs [14,17].

When studying SSR loci, genome-wide annotations commonly combine repeat motifs into complementary and overlapping DNA pairings [4], such that CA = AC = GT = TG, GA = AG = CT = TC, AT = TA, and GC = CG. In each case, the first two motifs of each set (e.g. CA/AC) represent cases where the longest perfect repeat begins with each of the two bases and the second pair of motifs (e.g. GT/TG) are the complementary base. Our approach counts each of the 12 dimer motifs independently and determines the maximum size of a perfect microsatellite repeat regardless of the starting base. Using these criteria, we observe a bias in the starting (and ending) nucleotide of dimer repeat loci. While the number of repeats from each of the two strands is necessarily equivalent, the starting base for each motif is significantly biased in all cases for both dimers and trimers (Table 1). For trimers, we examined the overall codon usage for *Daphnia pulex*, and did not see any correlation between motif class representation and codon usage (Table 1).

**Table 1:** Abundance and Starting Nucleotide Preference for homopolymer and dimer loci in D. pulex

Dimers									
**Motif Type**	**Count (obs)**	**End (exp)**	**p-value**	**Starting Pref**.	**Motif Type**	**Count (obs)**	**End (exp)**	**p-value**	**Starting Pref**.

TA	48814	42186	0	T	GC	8444	7623	1E-39	G
AT	35558	42186			CG	6802	7623		
									
GA	33951	31919	0	G/T	AC	33773	29999	0	A/T
AG	30185	31919			CA	26535	29999		
TC	40029	31919			TG	35249	29999		
CT	23511	31919			GT	24437	29999		
									

Trimers									

									
AAC+	2728	2821	1E-78	T/C	ACT	598	695	7E-15	G/T
ACA*	2431	2821			CTA+	734	695		
CAA*	3380	2821			TAC+	791	695		
GTT*	2339	2821			AGT	665	695		
TGT*	2657	2821			TAG+	564	695		
TTG*	3390	2821			GTA+	815	695		
									
AAG	5734	4486	0	T/A	AGC+	1839	2823	1E-226	C/G
AGA*	3657	4486			GCA*	2363	2823		
GAA*	4278	4486			CAG+	4131	2823		
CTT	3393	4486			GCT	3115	2823		
TCT	3692	4486			TGC+	2725	2823		
TTC+	6161	4486			CTG	2767	2823		
									
AAT*	4099	2937	5E-216	A	AGG+	971	1039	2E-18	G/T
ATA+	2260	2937			GGA*	1222	1039		
TAA*	2406	2937			GAG+	988	1039		
ATT*	3533	2937			CCT	905	1039		
TAT*	2233	2937			TCC	1207	1039		
TTA	3093	2937			CTC	940	1039		
									
ACC+	855	1089	4E-40	C/T	ATC	1153	1404	8E-46	T
CAC+	1057	1089			TCA*	1703	1404		
CCA*	1383	1089			CAT*	1342	1404		
GGT	1034	1089			GAT*	1452	1404		
GTG	921	1089			TGA	1661	1404		
TGG*	1285	1089			ATG*	1111	1404		
									
ACG	1229	1373	8E-11	G	CCG	703	742	1E-47	G
CGA	1406	1373			CGC+	519	742		
GAC+	1561	1373			GGC	916	742		
CGT	1325	1373			CGG	731	742		
TCG	1267	1373			GCG+	585	742		
GTC	1452	1373			GCC	995	742		

This table shows nonrandom starting nucleotides in both dimers and trimers. Motif type indicates largest possible repeat identified for each staggered SSR set. P-value calculated using Pearson's chi-square test for random expectation based upon observed and expected frequencies.

Preferential starting base is determined by the highest frequency SSR for the motif grouping. * indicate highly used codons, + indicate rarely used codons.

One explanation for the unexpected count differential between motifs in a similar grouping is the existence of a motif specific pattern of end base substitution, a phenomenon that has been previously observed in chicken microsatellites [18]. These observations may be informative with regard to the origin, or maintenance of SSR loci. To explore these patterns further, we analyzed variation at all SSR loci within the *D. pulex *genome assembly involving repeat number, incomplete insertion deletion events (indels), and base substitutions.

### SSR heterozygosity

Multiple models have been developed to explain the pattern of SSRvariation, such as the stepwise mutation model (SMM) [19], and more recent models that focus on a balance between the rate and pattern of length variation and base substitutions [20]. To evaluate the relative levels of these two processes, we analyzed the number of loci that were heterozygous for motif unit-length variation and non-repeat unit polymorphism (NRUPs). First, we identified all SSR loci that showed significant evidence of repeat length variation in the raw sequence data from which the genome was assembled (see methods). In *Daphnia pulex*, we were able to assay 6,062,268 of the 7,229,342 total SSRs for heterozygosity, of which 23,360 of the SSRs varied in length by at least one perfect repeat. Although smaller scaffolds may contain additional repeats, there is a increased possibility that these smaller scaffolds are either contaminated sequences or paralogous sequences, therefore we excluded them from the heterozygosity analysis.

**Figure 3:**
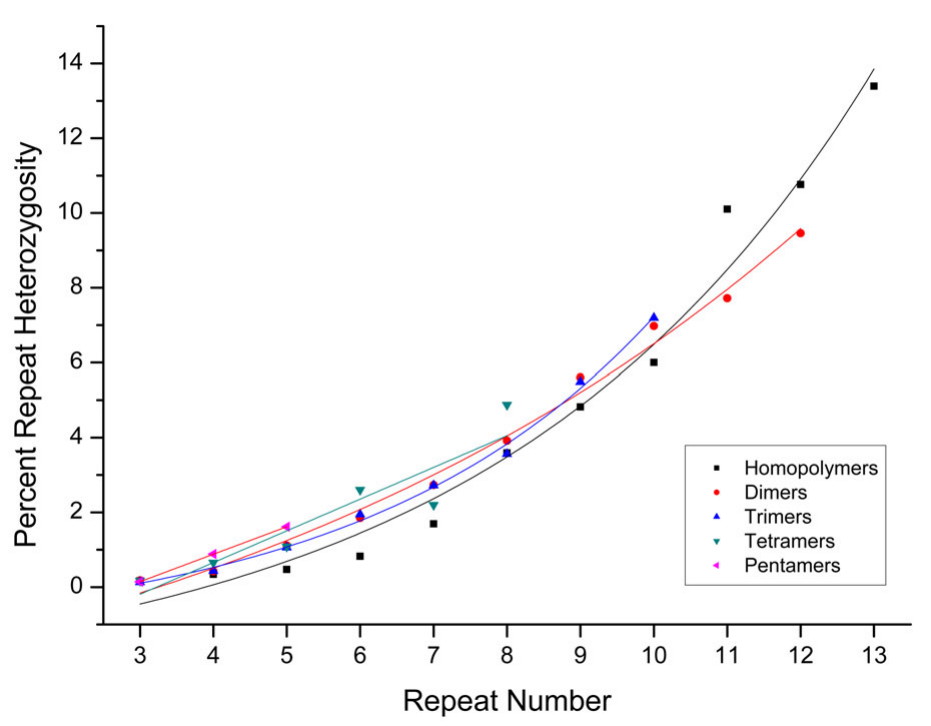
**Percent repeat heterozygosity for each motif size in *Daphnia pulex***. The X-axis is the repeat number found in the assembly. The Y-axis shows the percent of loci at that motif size that are heterozygous. Motif sizes best fit 1-3 an exponential curve while motif sizes 4 and 5 best fit a linear pattern. (Motifs 1-5 respectively: r^2 ^= 0.975, p < 1e-7; r^2 ^= 0.990, p < 1e-8; r^2 ^= 0.995, p < 1e-6; r^2 ^= 0.824, p < .01; r^2 ^= 0.999, p < .01).

For the five most abundant SSR motif sizes (1-5bp), we observe either an exponential or linear increase in repeat length heterozygosity as repeat number increases (Figure 3). Repeats with motif size greater than 5 were analyzed, however, due to the limited sample of large motifs, we were unable to discern any distinguishable pattern. The pattern observed in *D. pulex *for motifs with size 1-5bp is consistent with the previous observation in yeast and humans that mutation rate increases are correlated with increasing repeat number [7,17,21-23]. The observed pattern is unlikely to reflect SSR sequencing errors because while the frequency of sequencing errors does increase with the number of repeats, it is unlikely that the error will result in the variation of a perfect repeat unit.

**Figure 4:**
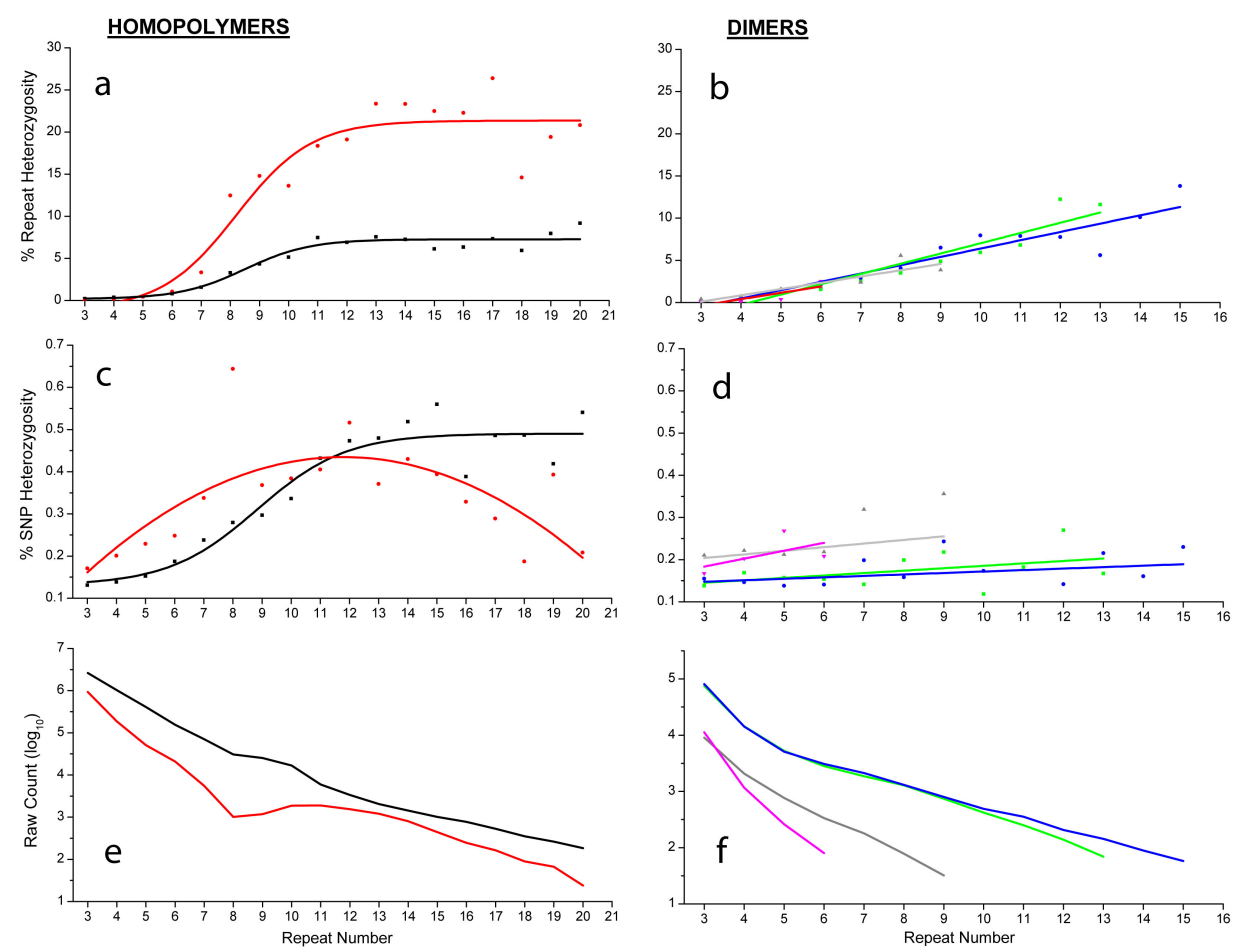
**Repeat heterozygosity, point mutation heterozygosity and raw counts for homopolymers (A, C, E) and dimers (B, D, F)**. Black lines and squares correspond to A/T homopolymers, red lines and squares correspond to C/G homopolymers. Colors corresponding to the dimers are green:AC/CA/GT/TG, blue:AG/GA/CT/TC, grey:AT/TA, magenta:CG/GC. (A) Percent repeat heterozygosity for homopolymers A/T (r^2^= 0.925, p < 1e-11) and C/G (r^2^= 0.899, p < 1e-10) with increases in heterozygosity highest between 7-11 repeats. C/G heterozygosity is approximately 3-fold higher than A/T heterozygosity. (B) Percent repeat heterozygosity for dimers with AC> AG> AT > CG (r^2 ^= 0.862, p < 1e-5; r^2 ^= 0.891, p < 1e-5; r^2 ^= 0.736, p < .01; r^2 ^= 0.539, p < .2). (C) Point mutation per nucleotide for homopolymers C/G (r^2 ^= 0.905, p < 1e-12) and A/T (r^2 ^= 0.446, p < .01), highest increase in polymorphism occurring at 7-11 repeats. (D) Percent point mutation/per base heterozygosity of microsatellites, AT/CG loci with higher proportions of point mutations than AC/AG types. (E) Raw genome counts of A/T and C/G homopolymers with frequency changes between 7-11 repeats. (F) Raw counts of dimers AC/AG/AT/CG.

We then further dissect repeat length heterozygosity, focusing on potential differences in heterozygosity among specific motif types. Due to the lack of heterozygotes in motifs longer than dimers, this analysis is limited to the two homopolymer and four dimer repeat motifs (Figure 4a and 4b). Homopolymers in *Daphnia pulex *exhibit increased repeat heterozygosity with increasing repeat number reaching a maximal rate of increase between 6 and 12 repeats (A/T r^2^= 0.925, G/C r^2^= 0.899). The G/C motif achieves a much higher frequency of heterozygosity (>20%) than A/T loci (~10%). The higher level of heterozygosity in G/C loci is consistent with direct estimates of the mutation rate using *Caenorhabditis elegans *mutation accumulation lines, where the G/C homopolymer mutation rate was ~20 fold greater than that for A/T HP loci [6], and consistent with previous experiments on *E. coli*, human, and yeast HPs which show a higher mutation rate of G/C HPs attributed to possible differences in base stacking properties during replication [24]. While it is possible that G/C loci with large numbers of repeats are being selectively excluded due to higher sequencing error rates, the fact that the A/T loci with lower levels of heterozygosity also show this plateau suggests that it may not simply be a sampling bias. If we assume that replication errors increase proportionately with an increase in repeat number, a plateau of A/T and G/C loci suggest that there may be a length threshold for differential repair activity, length-dependent counter mutation, or selection.

Similar to homopolymer repeats, dimer repeat heterozygosity increases with increasing repeat number (AC r^2 ^= 0.862; AG r^2 ^= 0.891, AT r^2 ^= 0.736, CG r^2 ^= 0.539) (Figure 4d). Our analysis indicates that the motifs AC and AG show the highest rate of increase in heterozygosity (1.22% and 0.99% per repeat unit) followed by AT and CG motifs (~0.75% per increase in repeat unit). In a pedigree study of forty-two microsatellite loci using six different *Drosophila melanogaster *populations, AC/AG repeats were shown to vary in repeat size at ~3-fold and ~1.5-fold more than AT repeats, with GC repeats not surveyed [12]. All other things being equal, both *Daphnia pulex *and *Drosophila melanogaster *have similar patterns of repeat variation such that the order of variation by motif sequence is AC/AG > AT > GC.

Studies in yeast and *C. elegans *have shown that the homopolymer mutation rate is ~100-fold greater than the mutation rate at dimer loci [3,7]. On a per locus basis, the percent heterozygosity of homopolymer repeats and dimer repeats are nearly identical in *Daphnia pulex *(+/- 0.5%), with the largest differences occurring at large repeats. Together, these observations suggest that the homopolymer and dimer mutation rates may be more similar in the *Daphnia *genome or that selection severely limits variation at homopolymer loci.

### Non-repeat Unit Polymorphisms

Non-repeat unit polymorphisms (NRUPs) are base substitutions or indels that interrupt the continuity of SSR locus and can shape the observed abundance of each repeat class (eg. CACATCACA is an interruption of a CA dimer with a T). It is extremely complicated to define and compare imperfect repeats, so for our analysis we identified SSR loci that are perfect repeats in the assembly, and subsequently looked for the existence of NRUPs in the reads aligned to each locus.

Examination of the NRUPs at HP loci (Figure 4c) reveals that as repeat number increases, the proportion of polymorphisms per nucleotide increases (C/G r^2 ^= 0.905, p < 1e-12; A/T r^2 ^= 0.446, p < .01). The rate of increase is particularly pronounced between 6 and 12 bases at HPs. We further evaluated the positions of NRUPs in a locus by comparing the site of polymorphism to the random expectation (Table 2). The result shows that at HP loci, point mutations occur at the beginning or end nucleotides at a higher frequency than expected, and the proportion increases with increasing repeat number, effectively shortening or lengthening the HP loci by a single unit. At smaller and more abundant repeat numbers (from 3-12 nucleotides), the number of end-point polymorphisms is substantial when compared to length variation, and point mutations may have a significant influence on homopolymer equilibrium.

**Table 2:** Position of the interruption in imperfect SSR.

	Homopolymers				
	
Repeat Size	End (obs)	Middle (obs)	End (exp)	Middle (exp)	p-value
3	15,005	50	10,037	5,018	0
4	5,793	1,392	3,593	3,593	0
5	2,549	1,168	1,487	2,230	5.E-277
6	1,309	735	681	1,363	1.E-190
7	810	492	372	930	5.E-159
8	447	294	185	556	3.E-109
9	459	253	158	554	7.E-162
10	350	285	127	508	2.E-108
11	178	191	67	302	1.E-50
12	119	167	48	238	1.E-29
13	65	121	29	157	1.E-13
14	45	107	22	130	7.E-08
15	34	77	15	96	8.E-08
16	18	43	8	53	6.E-05
17	12	40	6	46	1.E-02
18	6	28	4	30	2.E-01
19	3	23	3	23	9.E-01
20	6	15	2	19	5.E-03

	Dimers				
	
Repeat Size	End (obs)	Middle (obs)	End (exp)	Middle (exp)	p-value

3	1,588	641	1,486	743	5.E-06
4	332	208	270	270	9.E-08
5	121	83	82	122	2.E-08
6	65	61	42	84	1.E-05
7	58	53	32	79	3.E-08
8	47	29	19	57	1.E-13
9	39	30	15	54	7.E-12
10	17	10	5	22	2.E-08
11	11	7	3	15	2.E-06
12	9	7	3	13	2.E-05
13	7	4	2	9	9.E-06

This table shows the position of interruptions in simple sequence repeats. "End" indicates polymorphism at beginning or end of SSR loci, while middle variation indicates polymorphism occurs at interior positions (eg. Homopolymer with repeat length of three has two possible end nucleotide positions and one possible middle position. P-value is determined using Pearson's chi-square test for random expectation.

Similar to homopolymers, a significant end bias is found for NRUPs within dimer repeats (Table 2). At dimer loci with few repeats (less than 5), NRUPs outnumber unit length indels, while at loci with 6 or more repeats, unit length indels contribute to the majority of variation (Table 2). This pattern matches results found in a microsatellite study of the chicken genome, which also shows an end bias NRUP distribution within repeat arrays [18]. The propensity for end nucleotide polymorphisms at short repeats can lead to a SSR distribution characterized by species-specific mutation biases. Our analysis does not allow us to distinguish which of the heterozygous alleles is ancestral or derived and it would be interesting to understand if the rate of single nucleotide gains and losses are equivalent. A genome-wide SSR study involving two closely related populations with a recent out group would be required to determine how NRUPs effect the equilibrium of SSR length distribution in an organism.

For at least chicken and *D. pulex*, these observations suggest that the specific abundance of specific types of microsatellites within a genome reflects a dynamic balance between changes in repeat number and base substitution. When combined with unequal patterns of base substitution, these observations can explain the overabundance of specific starting and ending nucleotides in SSR loci (Table 1).

### Multi-step variation

**Figure 5:**
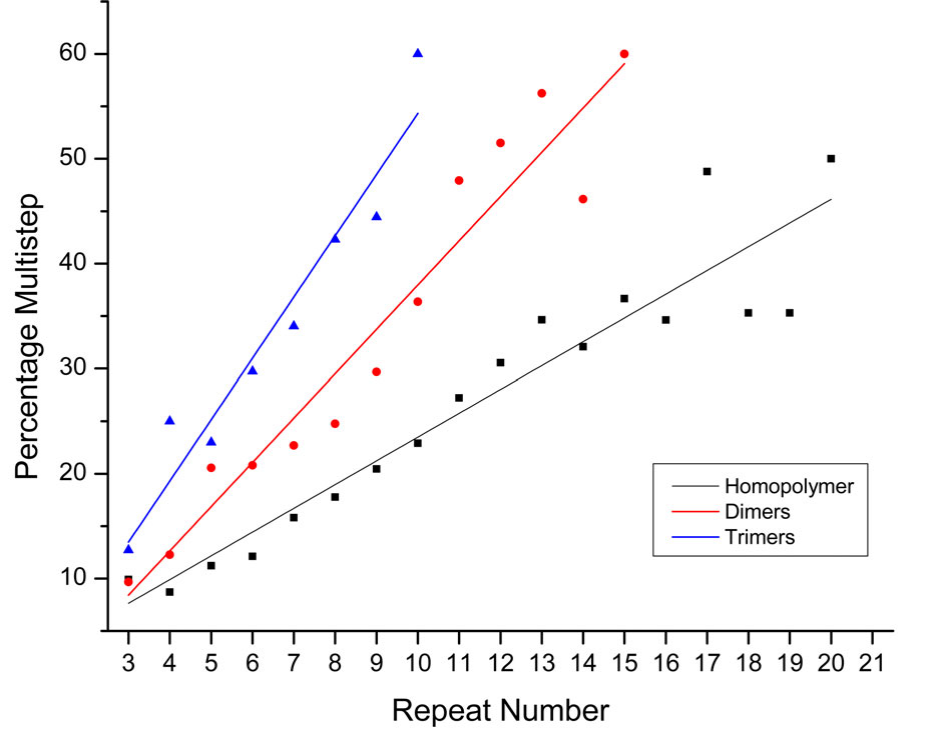
**Percentage of repeat heterozygosity that involves more than a single unit**. Homopolymers r^2 ^= 0.896, p < 1e-8; Dimers r^2 ^= 0.927, p < 1e-7; Trimers r^2 ^= 0.927, p < 1e-4.

Recent human microsatellite studies suggest that multi-step variation is a significant component of variation at larger SSR loci [25], deviating from the SSM model. Furthermore, researchers have shown that the directionality of multi-step mutations is length-dependent with a critical repeat number at which contractions are more frequent than expansions [26], while others suggest that a critical number does not exist [27]. In this experiment, we required that each multistep variant displayed a minimum of at least two full perfect repeats. Our analysis revealed two patterns. First, for all motif sizes, the proportion of heterozygotes that are multi-step increases with the number of repeats at a locus (Figure 5). Second, the rate of multi-step variation increases with increased motif size. Further analysis of individual homopolymer motif types showed that the fraction of multistep variation at A/T HP is much lower (12.14% or 2129/17544) than that of G/C HPs (27.4% or 972/3547) (Figure 5). Dimer loci also show a high frequency (~21.3% or 288/1353) of multistep polymorphisms, with AC motif showing the highest proportion multistep increase (0.0423) followed by AG (0.036) and AT (0.0301) (Figure 5). Direct estimates of dimer SSR loci in *D. pulex *and *C. elegans *show that 73% (173 of 237) of the variation at AC and AG loci larger than 13 repeats was multi-step variants [26]. A similar partition of our data reveals that 52% (12/23) of AC and AG dimer loci with greater than 13 repeats are multi-step differences. Although the experiments described above show that most of these multi-step changes are repeat length increases, our method of analysis does not allow us to polarize the differences between alleles. In any event, there appears to be a motif dependant correlation between repeat length and multi-step mutation change for SSR loci.

### Density of SSR Loci

Although the factors that affect the density of SSR loci within a genome remain to be clearly defined, the density of SSRs within genomes has been positively correlated with regional rates of recombination in *Drosophila melanogaster *[27], *Saccharomyces cerevisiae *[28], and *C. elegans *[2]. To evaluate the density of SSR loci in the *Daphnia *genome, we ranked the density of SSRs in each of the top 100 scaffolds ranging in size from 4.19 Mb to 0.47 Mb (Table 3a). Several scaffolds (68, 81, 83, 89) show a dearth of HP loci and scaffolds 66 and 98 have a paucity of SSRs with five and six base motifs respectively. While no scaffolds showed an overabundance of homopolymers, several scaffolds showed significantly high numbers of SSRs, in particular scaffolds 43, 74, and 76, which have an overabundance of repeats of motif sizes greater than 1 bp. Simple sequence repeats have been shown to play a role in gene regulation [1] and consequently would be in close proximity to genes. To test if these patterns are correlated with gene density, we compared SSR scaffold abundance to *Daphnia *gene predictions [15], and show that none of the overabundant scaffolds listed previously are either gene poor or gene rich (Table 3a). Results found in *C. elegans *[6] also show little correlation between gene density and SSR density.

**Table 3:** Gene content vs SSR abundance, and G/C content vs SSR heterozygosity

*Table 3a: Gene content vs SSR abundance*								
**Motif Size**								

**Abundance**	**1**	**2**	**3**	**4**	**5**	**6**	**2 to 6**	**Gene Content**

scaffold_18								+
scaffold_32		+			+		+	
scaffold_43		+					+	
scaffold_58					+			
scaffold_59						+		
scaffold_62					+			
scaffold_66					-			
scaffold_67						+		+
scaffold_68	+							
scaffold_74		+					+	-
scaffold_76		+		+		+		
scaffold_81	+							
scaffold_82	+				+			-
scaffold_84								-
scaffold_85						+		
scaffold_88					+			
scaffold_90			+	**+**				
scaffold_98						-		
								

*Table 3b: G/C content vs SSR heterozygosity*								

Motif Size								

Heterozygosity	1	2	3	4	5	6	2 to 6	G/C Content

scaffold_7						+		
scaffold_18					+			
scaffold_19						+		
scaffold_20					+			
scaffold_24				+		+		
scaffold_30	**+**	**+**	**+**				**+**	
scaffold_32				+				
scaffold_33					**+**			
scaffold_43		+						
scaffold_63						**+**		
scaffold_69						**+**		
scaffold_75					**+**			
scaffold_76		+				**+**	+	
scaffold_77			+				+	
scaffold_82								-
scaffold_90			**+**	**+**			+	+
scaffold_94				+				

This table shows motif sizes that are significantly greater than (+) or less than (-) the overall scaffold mean.

Motif Sizes that are significantly different (2 standard deviations) from the overall scaffold mean using Generalized Extreme Studentized Deviate (ESD) are bolded (p < 0.05, df = 98). Column 2 to 6 is the cumulative sum of motif sizes 2 to 6 (homopolymers excluded).

**Figure 6:**
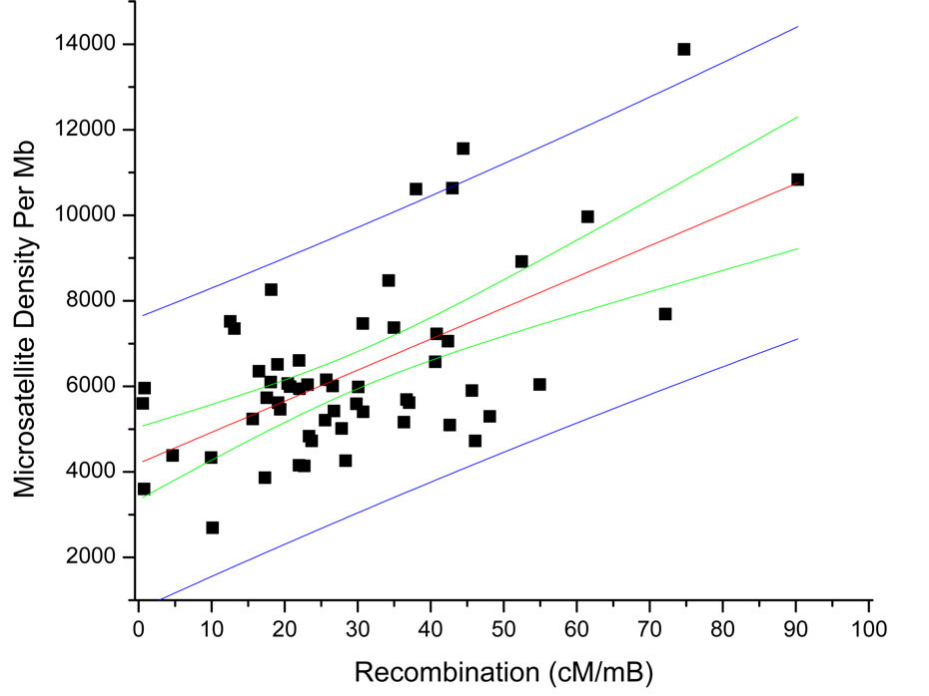
**Positive correlation between recombination (cM/mB) and microsatellite density per Mb**. 95% prediction interval represent the blue line, 95% confidence interval represent the green line (r^2 ^= 0.376, p < 1e-6).

**Figure 7:**
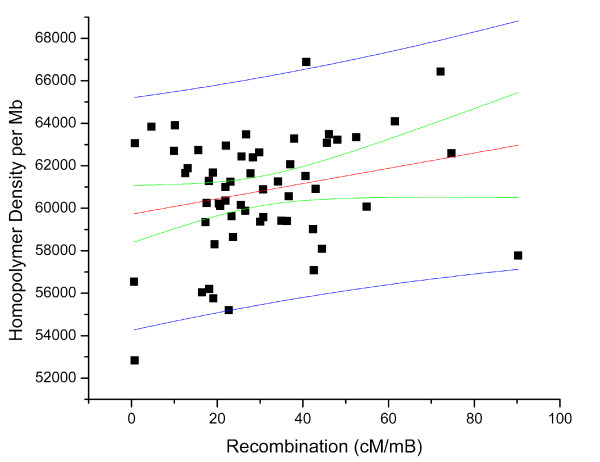
**Lack of correlation between recombination (cM/mB) and microsatellite heterozygosity**. 95% prediction interval represent the blue line, 95% confidence interval represent the green line (r^2 ^= 0.030, p > .1).

We then used a preliminary genetic map for *Daphnia pulex *[29] to test for correlations between SSR density and heterozygosity with rates of recombination within scaffolds. Based on 61 intervals in the genetic map that could be assigned to physical intervals in the top 100 scaffolds, we find that the density of microsatellite loci with motif sizes greater than 1bp show a significant positive correlation with the rate of recombination (Figure 6). This result is consistent with a yeast study showing a high frequency of microsatellite repeats near meiotic hotspots [28], and further supports role for SSR loci in the regulation of recombination. Both microsatellite repeats (repeats of motif size 2-6), and homopolymer repeats (single nucleotide repeats) correlate positively with recombination rate (Figure 7). Homopolymers have array sizes that are considerably larger than microsatellites, giving them properties that may influence recombination frequency.

**Figure 8:**
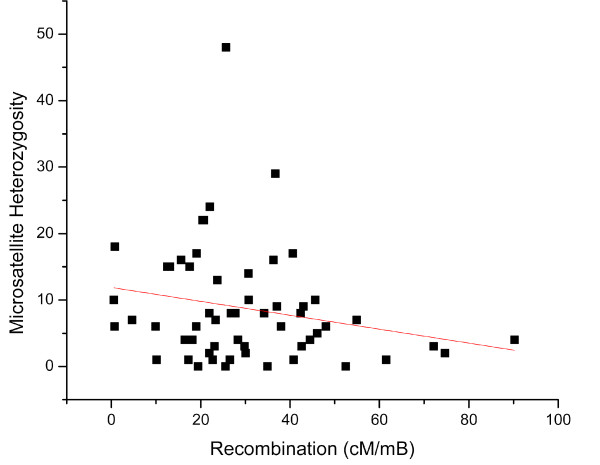
**Positive correlation between recombination (cM/mB) and microsatellite density per Mb**. 95% prediction interval represent the blue line, 95% confidence interval represent the green line (r^2 ^= 0.401, p < 0.08).

Within the same intervals, no correlation was observed between microsatellite heterozygosity and recombination in *Daphnia pulex *(Figure 8). A previous microsatellite survey in humans [30] also showed no significant correlation between microsatellite heterozygosity and recombination rate, however, the scale of the recombination intervals and their measurement often make such observations difficult. Although SSRs of all types are correlated with recombination rates, the level of SSR heterozygosity seems uninfluenced by recombination.

The distribution of heterozygosity at SSR loci can be shaped by selective sweeps, background selection and locus-specific differences in mutation rates. Many of these patterns may themselves be correlated. Based on our analysis, scaffolds 24, 30, 76, and 90 have multiple motif classes that are significantly more heterozygous than expected (Table 3b). Scaffold 90 also shows significantly higher G/C content (44%; average G/C = 41%), which is contradictory to suggestions of an evolved efficiency of MMR in G/C rich regions of eukaryotes [31].

### Conclusions

The *Daphnia pulex *genome sequence provides us with a unique opportunity to study genome-wide SSR patterns using whole genome shotgun reads. Although recent sequencing technologies have greatly improved sequencing throughput, these new technologies introduce multiple errors that are absent from WGS sequencing. Furthermore, the large read size of WGS sequencing allows for alignment to larger motif types that smaller read lengths cannot span. *Daphnia pulex *has undergone a minimal amount of inbreeding prior to sequencing, retaining high relative levels of assayable SSR heterozygosity that may be removed from heavily inbred genome sequencing projects.

SSRs, primarily microsatellites, are used commonly as genetic markers for population level studies. Our results show that in *Daphnia pulex*, levels of genome-wide SSR heterozygosity can not only vary with the length of motifs, but also by motif type in agreement with the studies of other organisms [18,22,27]. Our genome-wide results show that microsatellite loci with less than 6 repeats accumulate non-repeat unit polymorphisms at a greater rate than repeat length differences. In addition, AC/AG microsatellites accumulate repeat length differences at a greater rate than AT/GC microsatellites. Taken together, for *Daphnia pulex*, AC/AG repeats with a minimum of 6 repeats will provide the most resolution when used as genetic markers in *Daphnia *specific population level studies. Although the two available genome-wide microsatellite studies of fruit-fly [27] and chicken [18] display similar patterns of heterozygosity (highest AC/AG heterozygosity), microsatellites studies from additional taxa must be made before a broad recommendation for genetic marker motif type and repeat length can be made.

In our analysis of SSR loci in the genome of *Daphnia pulex *we describe both the catalogue of SSR sequences and several aspects of abundance and variation that are motif specific. Patterns that are motif specific include levels of heterozygosity, motif specific rates of repeat length variation, and motif specific patterns of NRUPs. Current models of simple sequence repeat evolution suggest that the abundance and variation at SSRs results from a balance of two opposing mutational forces. SSRs have a repeat number dependant rate of evolution resulting in the loss and gain of perfect repeat units. By contrast NRUPs disrupt repeat patterns and break down larger repeats into smaller ones. We also find a propensity for end nucleotide base substitution in SSRs, which was also reported in the chicken genome [18], suggesting that end nucleotide base substitution shapes the specific patterns of SSR abundance in multiple genomes. In addition to nucleotide end bias, we identify a significant difference in nucleotide starting preference for dimer and trimer classes.

## Methods

### Sequence data

The fasta sequences used in this study are from the *Daphnia pulex *genome project. The DOE Joint Genome Institute (JGI) and the *Daphnia *Genome Consortium (DGC) have sequenced 2,729,325 shotgun clones that result in 8.7 × coverage of the *Daphnia pulex *genome. This sequence has been assembled using the JAZZ assembler, and consists of 9,080 scaffolds, containing a total of 1,591,853 reads, and 227.1 Mb. In this assembly, 103 scaffolds represent the N50. The sequences can be downloaded at JGI http://www.jgi.doe.gov/Daphnia/) and the DGC http://wfleabase.org/. The fasta sequences used in Figure 2 were taken from http://www.flybase.org (*Drosophila melanogaster)*, http://www.wormbase.org (*Caenorhabditis elegans)*, and http://www.yeastgenome.org (*Saccharomyces cerevisiae*).

### Detection of simple sequence repeats

Programs written in PERL (available upon request) are used to count the number, length, location, and motif of all repeating motif size 1-100 bps in the *Daphnia *n50 scaffolds, with a minimum repeat number of 3 repeats. A greedy algorithm is applied to finding the repeats. Once the first repeat is found, the location is noted and back matching of the repeat is used to determine the length of the repeated motif. The program allows for no mismatches (all repeats are perfect repeats). Only the smallest motif in the repeat is counted, larger nested motifs are counted as the lowest common repeating motif (eg. GAGAGAGA is counted as 4 repeats of GA, not one repeat of GAGAGAGA or two repeats of GAGA). Each motif is defined by the first occurrence of a repeating nucleotide (eg. GA and AG are unique motifs). Because this is a greedy algorithm, motifs of the largest size are identified, regardless of sequence identity (eg. AAAGAGAAAGAG is counted as two repeats of AAAGAG, not two homopolymeric runs of AAA).

### Measuring Heterozygosity

To assay variation at SSR loci we used the AMOS reference assembler [32] to assemble the 8.7 × sequences (average read length = 774) to the JAZZ assembly at 90% identity to allow for an estimated 2-4% average sequence heterozygosity. This allows for heterozygous differences of between 6-8% (46-62 nucleotides). In order to remove paralogy, coverage depth at each position was limited to a maximum of 16 and a minimum of 4 inclusive. Loci that met these criteria through the entire repeat were extracted from the AMOS output and analyzed for indels of perfect repeats and point mutations. Variants that are greater than 1 repeat unit in length were categorized as multi-step variations. In order to make a heterozygous call, we required a minimum of 2 consensus reads showing the variation. There were 966758 sites that only had one variant read for repeat differences and 1675976 sites that only had one variant read for SNP differences. The total number of reads covering these sites were 9468378 and 15642919 reads respectively, leading to a read error rate of ~0.1 for both types. Loci that had more than two alleles were thrown out of the analysis. There were 1220 loci that displayed two or more heterozygotes SNP calls, and 277 loci that displayed more than two or more heterozygous repeat calls. (1497/6,062,268). The estimated frequency of paralogous loci in the dataset is 2.47e^-4.

## Authors' contributions

WS wrote the manuscript. AT, WS analyzed and interpreted data. AT, WS, RDB, WKT contributed to conception and design. ML and WKT participated in critical manuscript revision. All authors read and approved the final manuscript.
